# Supplementary material for: The preclinical pharmacological study on HX0969W, a novel water-soluble pro-drug of propofol, in rats
Source: PeerJ. 2020 Apr 16;8:e8922. doi: 10.7717/peerj.8922 (PMC7167245; doi:10.7717/peerj.8922)
Supplement: Supplemental Information 1 [file peerj-08-8922-s001.docx]

The sample preparation and quantitative method were detailed in the method section.

**1. LC–MS/MS method of HX0969W**

1.1.1. Speciﬁcity


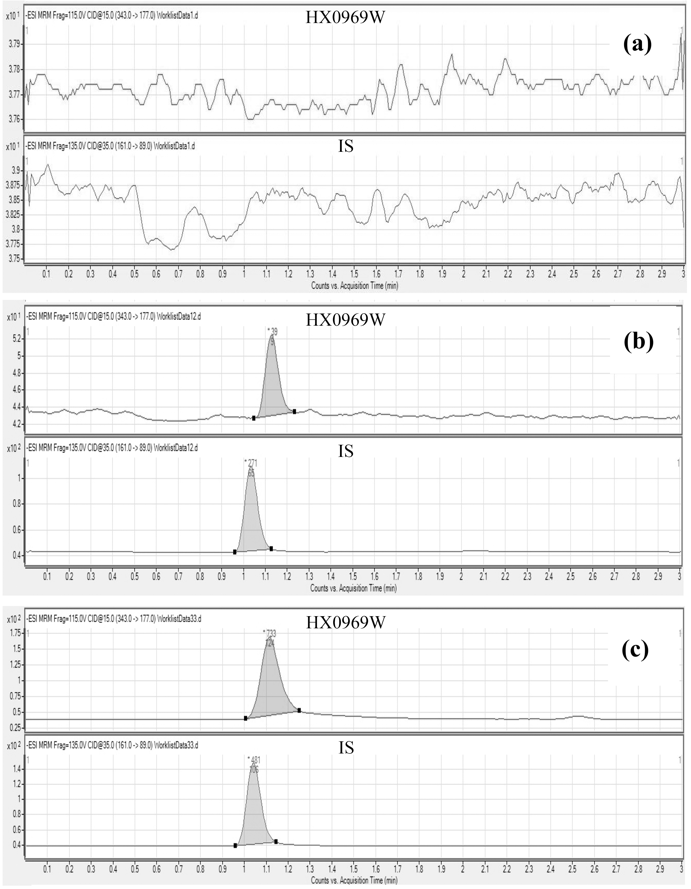


Representative chromatograms are presented, there were no interferences of HX0969W and internal standard (IS) from the matrixes (a: blank plasma, b: blank plasma with working solutions, c: the sample from the rat at 20 min after injected with 92.98 mg/kg HX0969W).

1.1.2. Linearity

The calibration curve showed a good linearity over the abovementioned calibration ranges (50 - 15,000 ng/mL). The typical equations for the calibration curves were as follows: y = 0.001925X + 0.01719. The correlation coefﬁcient was 0.9984. The lower limit of quantitation (LLOQ) of HX0969W was 50 ng/mL.

1.1.3. Precision and accuracy

| Spiked concentration  (ng/mL) | Intra-day | |  | Inter-day | |
| --- | --- | --- | --- | --- | --- |
|  | RE (%) | RSD (%) |  | RE (%) | RSD (%) |
| 50 | -14.18 | 6.56 |  | / | / |
| 150 | -7.77 | 1.61 |  | 1.62 | 8.23 |
| 1500 | -6.47 | 1.49 |  | -2.22 | 6.66 |
| 12000 | 4.94 | 0.97 |  | 5.32 | 5.23 |

Intra- and inter-day accuracy and precision were determined on the same day and on three different days, respectively, at three concentration levels of quality control (QC) samples in 5 replicates. All QC data were within ± 15% for the RE, and the RSD values were < 15%. The LLOQ samples were within ± 20% for the RE, and the RSD values were < 20%. The results demonstrated in table that the method was accurate, precise, and reproducible.

1.1.4. Matrix effect

| Spiked concentration  (ng/mL) |  | Matrix effect (%) | |
| --- | --- | --- | --- |
|  |  | HX0969W | IS |
| 150 |  | 94.56 | 88.13 |
| 1500 |  | 92.30 | 93.07 |
| 12000 |  | 88.93 | 90.83 |

The matrix effect of HX0969W and IS in plasma were found to be in the ranges of 88.93% to 94.56% for HX0969W and 88.13% to 93.07% for IS, respectively. The results obtained in the plasma indicated that there was no signiﬁcant interference with quantiﬁcation of HX0969W.

1.1.5. Extraction recovery

| Spiked concentration  (ng/mL) |  | Recovery (%) | |
| --- | --- | --- | --- |
|  |  | HX0969W | IS |
| 150 |  | 80.04 | 86.27 |
| 1500 |  | 87.35 | 86.71 |
| 12000 |  | 85.22 | 87.67 |

The low, middle, and high concentration of QC samples (LQC, MQC, and HQC, respectively) were assayed in sets of 5 replicates for the extraction recovery. As the table shown, the recovery was in the ranges of 80.04% to 87.35% for HX0969W and 86.27% to 87.67% for IS, respectively. Those results indicated that this method was consistent, precise, and repeatable.

1.1.6 Stability

| Storage conditions | spiked concentration | RE (%) | RSD (%) |
| --- | --- | --- | --- |
| Auto sampler  (8°C, 24 h) | 150 | -10.34 | 4.31 |
|  | 1500 | -7.96 | 3.20 |
|  | 12000 | 2.92 | 0.80 |
| Long term  (-20°C, 7 days) | 150 | -7.86 | 8.36 |
|  | 1500 | -13.88 | 1.20 |
|  | 12000 | -3.46 | 5.33 |

Stability was examined for QC samples at two different storage conditions (n = 5). The results showed that post-preparative samples were stable for 24 h in an autosampler at 8 °C. The data of long-term stability conﬁrmed that samples could be stored for at least 7 days at -20 °C.

1.1.7 Dilution integrity

Samples with concentrations exceeding the upper limit of quantitation (ULOQ) were diluted with a blank matrix to obtain a concentration within the calibration range. To validate the dilution effect, HX0969W in plasma at concentration of 10 × 10^5^ ng/mL were prepared (n = 5). All the samples were diluted 100-fold using blank rat plasma. After dilution, samples were prepared and determined. The percent deviations of the back-calculated concentrations from the theoretical values were -9.59% (RE) and 3.40% (RSD), respectively. The results of the dilution integrity showed that it had no inﬂuence on the accuracy of determination of sample concentrations.

**2. LC–MS/MS method of fospropofol disodium**

2.1.1. Speciﬁcity


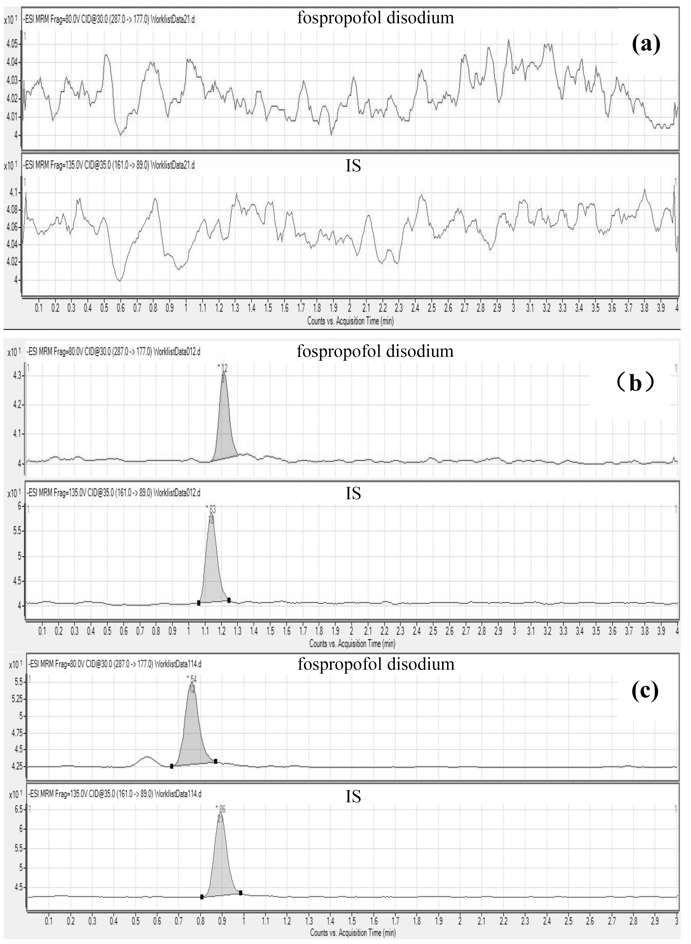


Representative chromatograms are presented, there was no interferences of fospropofol and IS from the matrixes (a: blank plasma, b: blank plasma with working solutions, c: the sample from rat at 20 min after injected with 87.32 mg/kg fospropofol).

2.1.2. Linearity

The calibration curve showed a good linearity over the abovementioned calibration ranges (120 - 15,000 ng/mL). The typical equations for the calibration curves were as follows: y = 0.0007733X - 0.002499. The correlation coefﬁcient was 0.9970. The LLOQ of fospropofol was 120 ng/mL.

2.1.3. Precision and accuracy

| Spiked concentration  (ng/mL) | Intra-day | |  | Inter-day | |
| --- | --- | --- | --- | --- | --- |
|  | RE (%) | RSD (%) |  | RE (%) | RSD (%) |
| 120 | 0.41 | 9.72 |  | / | / |
| 360 | -4.80 | 4.67 |  | -5.96 | 6.62 |
| 1500 | -3.05 | 2.42 |  | -8.04 | 5.35 |
| 12000 | 8.42 | 1.81 |  | -0.39 | 7.72 |

Intra- and inter-day accuracy and precision were determined on the same day and on three different days, respectively, at three concentration levels of QC samples in 5 replicates. All QC data were within ± 15% for the RE, and the RSD values were < 15%. The LLOQ samples were within ± 20% for the RE, and the RSD values were < 20%. The results demonstrated in table that the method was accurate, precise, and reproducible.

2.1.4. Matrix effect

| Spiked concentration  (ng/mL) |  | Matrix effect (%) | |
| --- | --- | --- | --- |
|  |  | fospropofol | IS |
| 360 |  | 89.85 | 89.72 |
| 1500 |  | 89.81 | 78.56 |
| 12000 |  | 86.21 | 81.94 |

The matrix effect of fospropofol and IS in plasma were found to be in the ranges of 86.21% to 89.85% for fospropofol and 81.94% to 89.21% for IS, respectively. The results obtained in the plasma indicated that there was no signiﬁcant interference with quantiﬁcation of fospropofol.

2.1.5. Extraction recovery

| Spiked concentration  (ng/mL) |  | Recovery (%) | |
| --- | --- | --- | --- |
|  |  | fospropofol | IS |
| 360 |  | 77.87 | 89.02 |
| 1500 |  | 78.05 | 90.44 |
| 12000 |  | 82.76 | 86.00 |

The LQC, MQC, and HQC samples were assayed in sets of 5 replicates for the extraction recovery. As the table shown, the recovery was in the ranges of 77.87% to 90.44% for fospropofol and 86.00% to 90.44% for IS, respectively. Those results indicated that this method was consistent, precise, and repeatable.

2.1.6 Stability

| Storage conditions | Spiked concentration | RE (%) | RSD (%) |
| --- | --- | --- | --- |
| Auto sampler  (8 °C, 24 h) | 360 | 7.90 | 4.11 |
|  | 1500 | -9.65 | 5.57 |
|  | 12000 | -4.17 | 5.84 |
| Long term  (-20°C, 7 days) | 360 | 7.39 | 1.05 |
|  | 1500 | 6.99 | 2.69 |
|  | 12000 | 13.74 | 7.57 |

Stability was examined for QC samples at two different storage conditions (n = 5). The results showed that post-preparative samples were stable for 24 h in an autosampler at 8 °C. The data of long-term stability conﬁrmed that samples could be stored for at least 7 days at -20 °C.

2.1.7 Dilution integrity

Samples with concentrations exceeding the ULOQ were diluted with a blank matrix to obtain a concentration within the calibration range. To validate the dilution effect, fospropofol in plasma at concentration of 10 × 10^5^ ng/mL were prepared (n = 5). All the samples were diluted 100-fold using blank rat plasma. After dilution, samples were prepared, and then determined. The percent deviations of the back-calculated concentrations from the theoretical values were -8.68% (RE) and 3.06% (RSD), respectively. The results of the dilution integrity showed that it had no inﬂuence on the accuracy of determination of sample concentrations.

**3. HPLC- fluorescence method of propofol**

3.1.1. Speciﬁcity


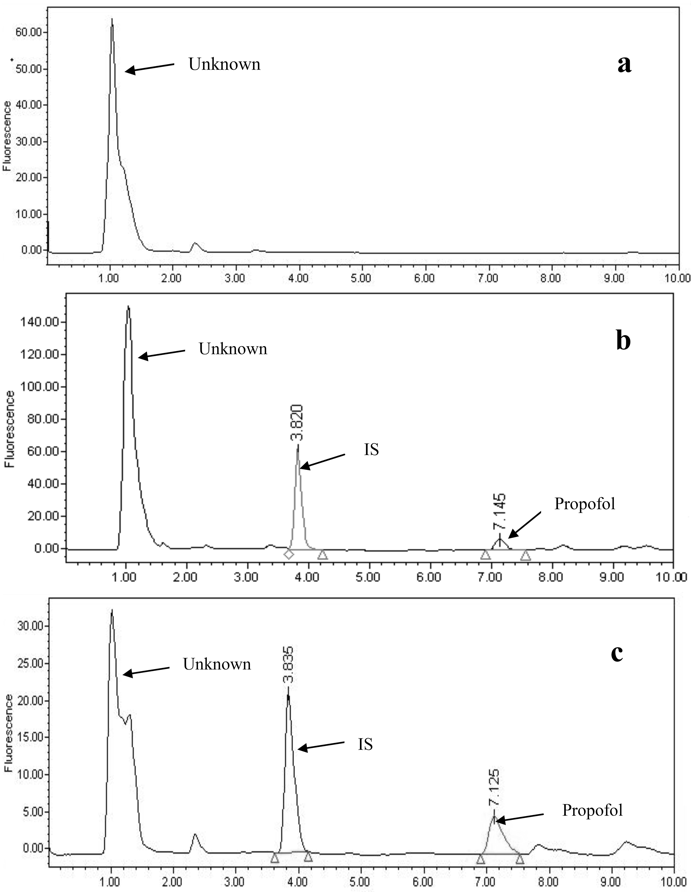


Representative chromatograms are presented, there was no interferences of propofol and IS from the matrixes. (a: blank plasma, b: blank plasma with working solutions, c: sample from rat injected with 9.64 mg/kg propofol after 20 min)

3.1.2. Linearity

The calibration curve showed a good linearity over the abovementioned calibration ranges (50 - 80,000 ng/mL). The typical equations for the calibration curves were as follows: y = 0.0002765X - 0.0007835. The correlation coefﬁcient was 0.9993. The LLOQ of propofol was 50 ng/mL.

3.1.3. Precision and accuracy

| Spiked concentration  (ng/mL) | Intra-day | |  | Inter-day | |
| --- | --- | --- | --- | --- | --- |
|  | RE (%) | RSD (%) |  | RE (%) | RSD (%) |
| 50 | 11.68 | 5.12 |  | / | / |
| 100 | 4.52 | 5.41 |  | 2.98 | 6.22 |
| 2000 | 2.09 | 3.86 |  | 1.15 | 5.33 |
| 60000 | 9.31 | 2.98 |  | 3.75 | 6.31 |

Intra- and inter-day accuracy and precision were determined on the same day and on three different days, respectively, at three concentration levels of QC samples in 5 replicates. All QC data were within ± 15% for the RE, and the RSD values were < 15%. The LLOQ samples were within ± 20% for the RE, and the RSD values were < 20%. The results demonstrated in table that the method was accurate, precise, and reproducible.

3.1.4. Extraction recovery

| Spiked concentration  (ng/mL) |  | recovery (%) | |
| --- | --- | --- | --- |
|  |  | propofol | IS |
| 100 |  | 89.27 | 80.42 |
| 2000 |  | 83.50 | 78.84 |
| 60000 |  | 86.81 | 77.74 |

The LQC, MQC, and HQC samples were assayed in sets of 5 replicates for the extraction recovery. As the table shown, the recovery was in the ranges of 83.50% to 89.27% for propofol and 77.74% to 80.42% for IS, respectively. Those results indicated that this method was consistent, precise, and repeatable.

3.1.5. Stability

| Storage conditions | Spiked concentration | RE (%) | RSD (%) |
| --- | --- | --- | --- |
| Auto sampler  (30 °C, 24 h) | 100 | 10.33 | 4.63 |
|  | 2000 | -0.28 | 3.45 |
|  | 60000 | 6.30 | 7.24 |
| Long term  (-20°C, 7 days) | 100 | 4.81 | 2.88 |
|  | 2000 | -1.66 | 1.29 |
|  | 60000 | 2.70 | 2.36 |

Stability was examined for QC samples at two different storage conditions (n = 5). The results showed that post-preparative samples were stable for 24 h in an autosampler at 30 °C. The data of long-term stability conﬁrmed that samples could be stored for at least 7 days at -20 °C.
